# Inguinal Endometriosis: An Unusual Cause of Groin Pain

**DOI:** 10.4274/balkanmedj.galenos.2020.2020.2.105

**Published:** 2020-08-11

**Authors:** Hirohisa Fujikawa, Yuya Uehara

**Affiliations:** 1 Department of Medical Education Studies, International Research Center for Medical Education, Graduate School of Medicine, The University of Tokyo, Tokyo, Japan; 2 Department of Internal Medicine, Suwa Central Hospital, Nagano, Japan; 3 Department of Surgery, Suwa Central Hospital, Nagano, Japan

A previously healthy 42-year-old woman presented to the hospital with a 1-year history of right groin pain, which did not fluctuate with the menstrual cycle. We suspected an inguinal hernia or lymphadenopathy, but a right inguinal ultrasound revealed a mixed-echo mass with intralesional vascular flow (Figure 1A). Pelvic magnetic resonance imaging (MRI) revealed a mass that showed high intensity on T1- and T2-weighted images (Figure 1B, 1C). The mass was located at the apex of the inguinal hernia sac. Subsequently, we performed a surgical biopsy and resected the mass with a wide surgical margin, considering the possibility of malignancy and recurrence. A histological examination revealed the presence of endometriotic lesions and hemosiderin-laden macrophages (Figure 1D). Therefore, we diagnosed the patient with inguinal endometriosis. The patient was relieved from pain and has not experienced recurrence. The patient’s consent was obtained.

Endometriosis is a common and chronic and benign gynecological disorder that is estrogen-dependent. It is defined as the presence of endometrial glands and stroma outside the endometrial cavity (1). While the most common sites for endometriosis are within the pelvis, uncommon locations include the intestines, surgical scars, diaphragm, umbilicus, and groin.

Inguinal endometriosis is rare, with an incidence of 0.3%-0.6% in all endometriosis cases (2,3). It presents with common symptoms such as the presence of an inguinal mass or pain. MRI is a useful diagnostic tool that allows the detection of iron in the hemosiderin deposits within an endometrioma. Therapeutic options include hormonal therapy and/or complete surgical excision to avoid spillage and prevent its recurrence. While pelvic endometriosis usually causes cyclical pain that is exacerbated during menstruation, inguinal endometriosis frequently presents with a constant pain, which is not associated with the menstrual cycle (4). Therefore, inguinal endometriosis can mimic other common diseases such as hernia, lymphadenopathy, abscess, and cancer (4), and patients may visit multiple departments, including internal medicine, surgery, and gynecology, before the diagnosis of inguinal endometriosis. The inguinal involvement of endometriosis should be considered in the differential diagnosis of a painful inguinal mass in women of reproductive age.

## Figures and Tables

**Figure 1:**
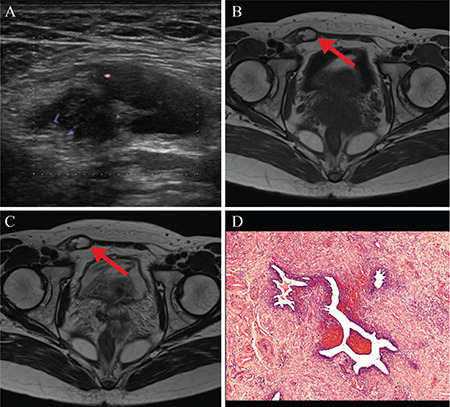
**A-D. Images of the patient:** (A) Inguinal ultrasound depicted a mixed-echo mass with intralesional vascular flow in the right groin area. (B) Pelvic magnetic resonance imaging T1-weighted axial image revealed a high-signal intensity mass in the right inguinal area (arrow). (C) Pelvic magnetic resonance imaging T2-weighted axial image demonstrated a high-signal intensity mass in the right inguinal area (arrow). (D) Histological evaluation of the groin mass showed the presence of endometriotic lesions and hemosiderin-laden macrophages (hematoxylin and eosin stain, original magnification 40x).
